# Genomic evidence for three distinct species in the *Erebia manto* complex in Central Europe (Lepidoptera, Nymphalidae)

**DOI:** 10.1007/s10592-023-01501-w

**Published:** 2023-01-10

**Authors:** Amanda Jospin, Yannick Chittaro, Daniel Bolt, David Demergès, Kevin Gurcel, Jürgen Hensle, Andreas Sanchez, Christophe Praz, Kay Lucek

**Affiliations:** 1 Laboratory of Functional Ecology, Institute of Biology, University of Neuchâtel, Rue Emile-Argand 11, 2000 Neuchâtel, Switzerland; 2 Info Fauna, Avenue de Bellevaux 51, 2000 Neuchâtel, Switzerland; 3 Domat/Ems, Switzerland; 4 Conservatoire d’espaces Naturels de Lorraine, 20 Chemin de L’école Des Xettes, 88400 Gérardmer, France; 5 Rumilly, France; 6 Teningen, Germany; 7 Department of Environmental Sciences, University of Basel, Schönbeinstrasse 6, 4056 Basel, Switzerland; 8 Biodiversity Genomics Laboratory, Institute of Biology, University of Neuchâtel, Rue Emile-Argand 11, 2000 Neuchâtel, Switzerland

**Keywords:** Conservation, Lepidoptera, Cryptic species, Species delimitation, RAD-sequencing

## Abstract

A problem to implement conservation strategies is that in many cases recognized taxa are in fact complexes of several cryptic species. Failure to properly delineate species may lead to misplaced priorities or to inadequate conservation measures. One such species complex is the yellow-spotted ringlet *Erebia manto*, which comprises several phenotypically distinct lineages, whose degree of genomic isolation has so far not been assessed. Some of these lineages are geographically restricted and thus possibly represent distinct units with conservation priorities. Using several thousand nuclear genomic markers, we evaluated to which degree the *bubastis* lineage from the Alps and the *vogesiaca* lineage from the Vosges, are genetically isolated from the widespread *manto* lineage. Our results suggest that both lineages are genetically as strongly differentiated from *manto* as other taxonomically well separated sibling species in this genus from each other, supporting a delineation of *bubastis* and *vogesiaca* as independent species. Given the restricted and isolated range of *vogesiaca* as well as the disjunct distribution of *bubastis,* our findings have significant implication for future conservation efforts on these formerly cryptic species and highlight the need to investigate the genomic identity within species complexes.

**Supplementary Information:**

The online version contains supplementary material available at 10.1007/s10592-023-01501-w.

## Introduction

Insect diversity and biomass are declining at accelerated rates in Europe and elsewhere (Hallmann et al. 2017, Seibold et al. 2019, van Klink et al. 2020), and insect conservation has become a key topic in conservation biology in the last decades (Dunn 2005; Forister et al. 2019). Poorly known life histories or geographic distributions combined with often challenging identification and unresolved taxonomy may hamper conservation efforts for invertebrates more than vertebrates. An incorrect taxonomy that amalgamates species complexes into single taxa may for example lead to erroneous prioritization in conservation or to inadequate conservation measures (Ceballos et al. 2017). This situation can occur when the overall taxon is classified as being of low concern for conservation due to the broad occurrence of the entire species complex, or if conservation measures are based on ecological assumptions from the entire species complex and do not account for  ecologically more specialized cryptic species (Bickford et al. 2007). It is therefore important to examine such species complexes and to delineate units for conservation efforts.

Across Europe, almost 500 species of butterflies have been recognized (Wiemers et al. 2018), many of which are declining in their population sizes and/or distribution ranges (Warren et al. 2021). Numerous taxa comprise different subspecies or lineages (Settele et al. 2008), which may moreover hybridize to varying degrees upon secondary contact (Descimon and Mallet 2009). The genus *Erebia* is known for its cryptic diversity (Sonderegger 2005; Descimon and Mallet 2009) and also includes “geographical replacement species”, *i.e.* species or lineages that replace each other by their vicariant sibling in parts of their range (Vodă et al. 2015). The taxonomic status of such species or lineages often remains ambiguous because they are possibly weakly isolated and some level of hybridization is still possible.

For *Erebia*, the degree of differentiation upon secondary contact between closely related species or lineages varies (Sonderegger 2005). For example, subspecies of *Erebia euryale* form narrow contact zones with phenotypic intermediates (Cupedo 2014; Cupedo and Doorenweerd 2022). This contrasts with the *E. tyndarus*-group, where geographic lineages represent reproductively isolated species with nearly interrupted gene flow. In particular, *E. cassioides* and *E. tyndarus* are parapatric in the Alps, and a third species, the very restricted *E. nivalis*, is found in range sympatry with the other two. Recent genetic studies showed that F1 hybrids between *E. cassioides* and *E. tyndarus* very rarely occur at their narrow zone of secondary contact, but no F2 hybrids have been reported among these three taxa, which exhibit limited levels of admixture, suggesting near complete reproductive isolation, consistent with distinct species (Gratton et al. 2016; Lucek et al. 2020; Augustijnen et al. 2022). Similar cases of strict parapatry without morphological intergradation also occur in other *Erebia*, leading to fragmented distributions and raising conservation concerns (Sonderegger 2005). This situation is observed in *E. sudetica* and *E. melampus*, two taxa that have non-overlapping distributions in Europe. In Switzerland, the former is limited to a few, small areas where *E. melampus* is absent.

**Fig. 1:**
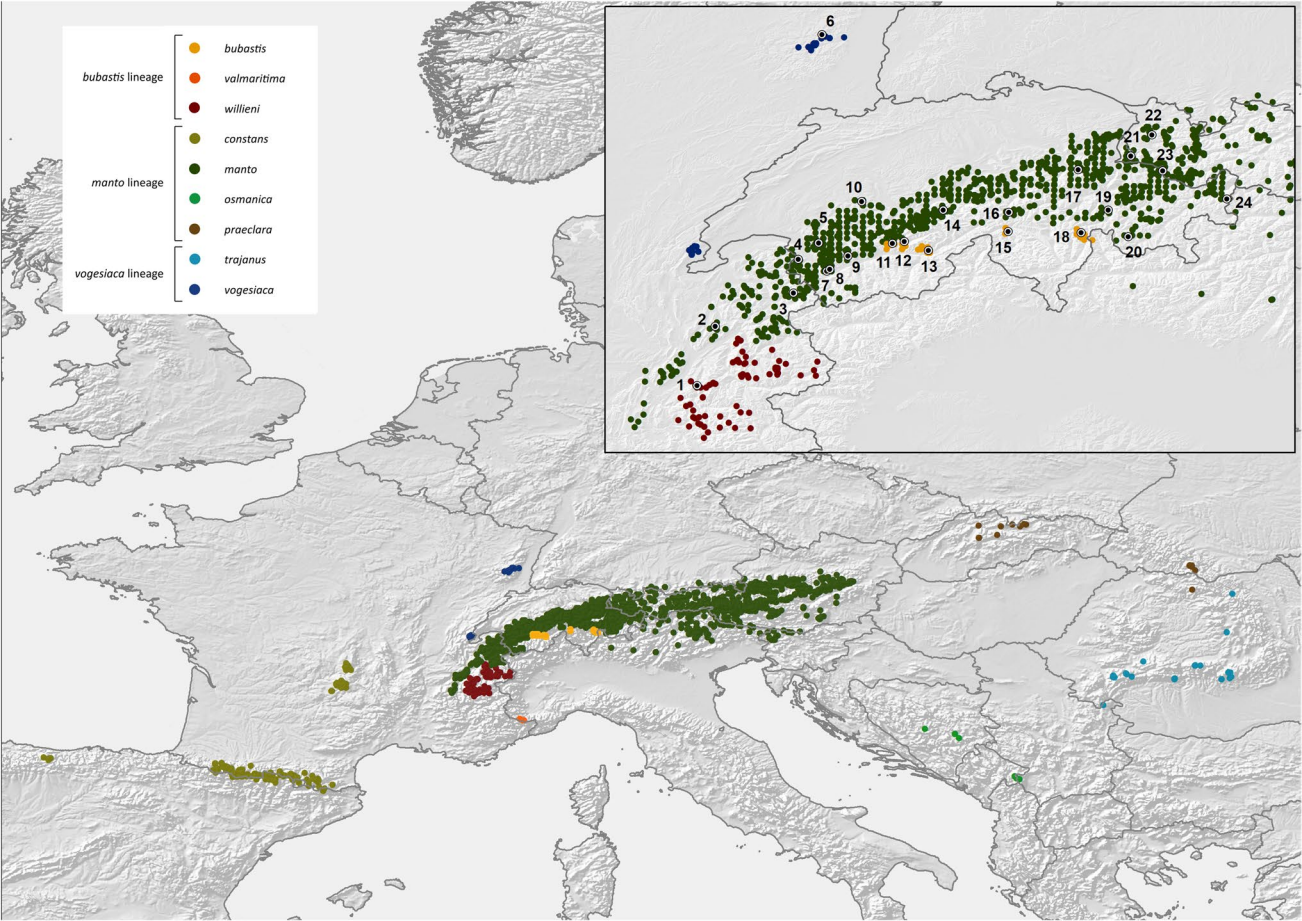
Distribution of the three lineages of the *Erebia manto* species complex across Europe. Each dot depicts an individual occurrence based on data from GBIF.org (accessed 16th of March 2022 with additional data from Lelo 2000; Burnaz 2008; Sanz et al. 2017; Jakšic 2019). A few doubtful occurences, as well as a few occurrences in the French Alps south of Grenoble, which could not be attributed unambiguously to one subspecies, have been omitted. Colors depict the different recognized *E. manto* subspecies (see Cupedo & Doorenweerd 2020). The inset depicts the Alps with numbers highlighting the populations sampled (see Supplementary Information 1 for details). The attribution to some occurrences to the different subspecies have been made following Cupedo (1997), Schmitt et al. (2014) and Cupedo & Doorenweerd (2020). For simplicity, *manto* and *mantoides* are merged and treated as "*manto*" given the uncertainties regarding the type locality of *manto* (see Cupedo & Doorenweerd 2020). Map sources: European Comission GISCO: https://ec.europa.eu/eurostat/web/gisco; EU-DEM https://www.eea.europa.eu/data-and-maps/data/eu-dem

**Fig. 2:**
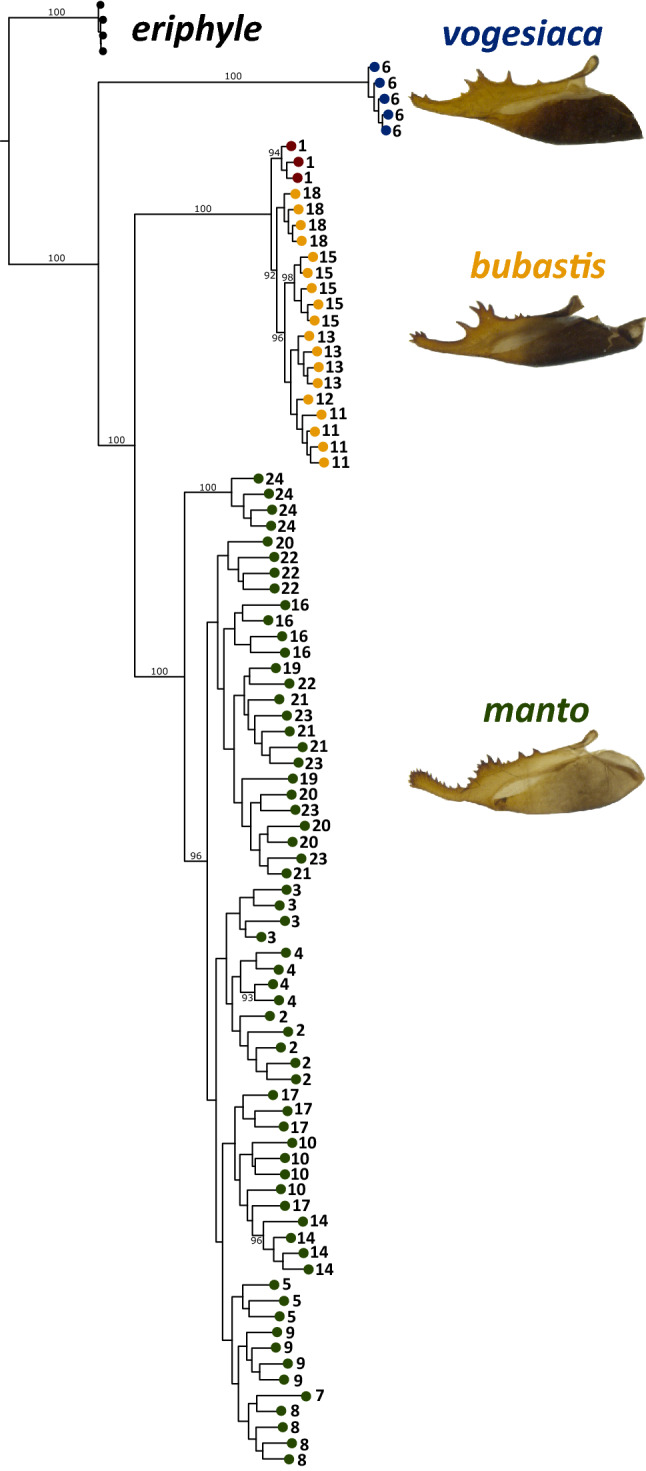
Phylogram artificially rooted on the node that separates *E. eriphyle* from the *E. manto* complex comprising *manto*, *bubastis* and *vogesiaca*. Numbers depict bootstrap support for nodes with > 90% support. Colors indicate taxa. Numbers at the terminal branches indicate sampling location as given in Fig. 1. For each *E. manto* lineage, a picture of the male genital valve is given

The yellow-spotted ringlet (*Erebia manto* complex) is a locally common butterfly with an insular-like distribution across Europe (Fig. 1), occurring along disconnected mountain ranges from northern Spain to the Carpathians, including the Alps and the Vosges (Schmitt et al. 2014; Kudrna et al. 2015). This disjunct distribution together with geographic variation in morphology has triggered the description of many allopatric *E. manto* subspecies in the past (Cupedo 1997; Cupedo and Doorenweerd 2020). The taxonomic status of these subspecies, is, however, debated (Sonderegger 2005; Schmitt et al. 2014; Cupedo and Doorenweerd 2020). Morphological and allozyme studies have indicated that the current distribution of the *E. manto* complex is likely shaped by post-glacial recolonization events from distinct glacial refugia, suggesting that part of the described diversity within the *E. manto* complex may therefore be old and predate the last glacial cycle (Cupedo 1997; Schmitt et al. 2014). Based on a detailed morphological study, Cupedo (1997) proposed to recognize the *manto*, the *bubastis* and the *vogesiaca* lineages, which substantially differ in genital structure. In particular, *manto* has substantially more spines on the valve than *bubastis*, while *vogesiaca* shows an intermediate phenotype (Cupedo 1997; Sonderegger 2005; Cupedo and Doorenweerd 2020; Fig. 2). Such marked differentiation in genital morphology in butterflies and in the genus *Erebia* in particular, is frequently used to delineate species (Warren 1936; Sonderegger 2005). Difference in male genital morphology may act as a reproductive barrier through lock-and-key mechanisms that reduce or prevent interspecific gene flow (Hollander et al. 2018). However, the lineages of the *manto* complex have so far mostly been treated as infraspecific units. Cupedo (1997) suggested that the phenotypic differences among the three lineages were as important as those observed between other *Erebia* species, but refrained from formally recognizing them as distinct species because evidence from cross experiments was lacking. By contrast, Cupedo and Doorenweerd (2020) recommended to treat the three lineages as conspecific due to one event of mitochondrial introgression between *manto* and *bubastis*. As far as it is known, the three lineages currently never occur in sympatry. While the *manto* lineage is widespread throughout Europe, the distribution of the other two lineages is more restricted. The *vogesiaca* lineage, in particular, is found in the Vosges (ssp. *vogesiaca*), possibly with an isolated population in the French Jura (Cupedo and Doorenweerd 2020), and in the Carpathian Mountains (ssp. *trajanus*). The *bubastis* lineage is restricted to narrow areas in the southwestern (ssp. *valmaritima*), western (ssp. *willieni*) and central Alps (ssp. *bubastis*), where it shows a disjunct distribution intermixed with populations of the *manto* lineage, which is widely distributed in Europe, including in the Alps (Fig. 1; Sonderegger 2005; Cupedo and Doorenweerd 2020).

The status of the lineages of the *manto* complex has only partially been examined using genetic data. Based on allozymes, Schmitt et al. (2014) demonstrated the presence of six genetic clusters in Europe, one in the Pyrenees and the Massif Central (ssp. *constans* belonging to the *manto* lineage), one in the Vosges (*vogesiaca*), three in the Alps and the Slovakian Carpathians (including ssp. *mantoides* and ssp. *manto*; both are subsequently merged and referred to as ssp. *manto* for simplicity due to uncertainties regarding the type locality of *manto*; see Cupedo and Doorenweerd 2020), and one in the Carpathians mountains (ssp. *trajanus*). Unfortunately, no *bubastis* sample was included, preventing conclusions on the status of the *bubastis* lineage. They nonetheless showed that the divergence between the *vogesiaca* and the other forms were as deep as those between the *manto* complex and the closely-related species *E. eriphyle*. These results suggested that the separation between the lineages of the *manto* complex could be far more divergent than initially assumed. More recent research based on mitochondrial DNA indicate that *manto* and *bubastis* exhibit minimal, but constant differences in their barcode sequences, except for one site in the French Alps; the *manto* lineage was though paraphyletic with respect to *bubastis* (Litman et al. 2018; Cupedo and Doorenweerd 2020). While mitochondrial barcodes may often suffice to delineate European butterfly species (Dincă et al. 2021), they may not have the resolution to do so in evolutionary young species or in the case of cytonuclear discordance as a result of incomplete lineage sorting and/or introgression (Toews and Brelsford 2012; Gueuning et al. 2020). Here, we used restriction-site associated DNA (RAD) markers to study the genetic relationship within part of the *manto* complex. We focused on the Alps and the Vosges and first assessed if and to which degree *bubastis* and *vogesiaca* may be genetically isolated from *manto*. Because the Alps were often recolonized by different glacial lineages of *Erebia* (Schmitt et al. 2006, 2016; Lucek et al. 2020), we further tested for genetic differentiation within *manto* and *bubastis* across the Alps. We discuss our findings for the *manto* complex in the broader context of *Erebia* systematics and their implications for conservation.

## Methods

### Sampling

We included a total of 95 butterflies collected during summer 2019 encompassing specimens of the three Central European *E. manto* lineages (following Cupedo and Doorenweerd 2020): *manto* (N = 65), *bubastis* (N = 21) and *vogesiaca* (N = 5). We further refer to these lineages simply as *manto*, *bubastis* and *vogesiaca* respectively. Sampling was conducted at 24 sites that were at least 3 km apart from each other and covered the Alps and the Vosges mountain ranges (Fig. 1; see Supplementary Information 1 for details). At each site, we collected one to six individuals (Supplementary Information 1). For *bubastis*, we specifically sampled the three locations in the Swiss Alps for which the presence of *bubastis* is documented (Sonderegger 2005; Litman et al. 2018). We further included a disjunct population of the *bubastis* lineage from the French Alps. This population belongs to the subspecies *willieni* and was collected at its type locality, located 180 km south-west from the nearest Swiss *bubastis* population (Cupedo 1997; Fig. 1); *manto* populations are found between this French and the Swiss *bubastis* populations. We also included four individuals of the closely related species *E. eriphyle* as an outgroup for our phylogenomic analysis. All individuals were captured with an insect net, their bodies stored in 100% ethanol and the wings kept separately. We a priori assigned each individual to an *E. manto* lineage based on geography combined with wing morphology for females and genital morphology for males. DNA extractions were performed using the Qiagen Dneasy Blood and Tissue kit (Qiagen, Zug, Switzerland) from the thorax. Wings and abdomens are deposited at the Natural History Museum of Neuchâtel, Switzerland.

Because *manto* and *bubastis* have been suggested to primarily occur on calcareous and siliceous substrates, respectively (Sonderegger 2005), we visualized their occurrence across the Swiss Alps in regard to broad scale geological substrate. For this we obtained location data with a precision of  < 100 m from the database of the Swiss zoological record centre (www.infospecies.ch) on the 2^nd^ of April 2022 for a total of 3886 *E. manto* and 81 *E. bubastis*. We further extracted the lithological-petrographic information for each occurrence based on geological layers of Switzerland (https://opendata.swiss/en/dataset/lithologisch-petrografische-karte-der-schweiz-gesteinklassierung-1-500000).

### Genetic data processing

We genotyped all 95 individuals using single-end restriction-site associated DNA (RAD) sequencing with the restriction enzyme *Sbf*I. Library preparation and sequencing on one Illumina HiSeq 4000 lane was outsourced to Floragenex (Portland, OR, USA).

We filtered all obtained genomic data following Lucek et al. (2020), *i.e.*, we only retained reads with an intact *Sbf*I restriction site, followed by de-multiplexing and barcode-trimming with *process_radtags* from Stacks 1.48 (Catchen et al. 2013). Using the FASTX toolkit (http://hannonlab.cshl.edu/fastx_toolkit/), we then removed reads containing bases with a Phred quality score < 10 or more than 5% of base pairs with quality < 30. This approach yielded ~ 370 million high quality reads in total for our analysis. In a next step, we mapped the reads of each individual against a genome assembly of *E. cassioides* with BWA MEM 0.7.17 (Li 2013) and genotyped all specimens with BCFtools 1.10.2 (Danecek and McCarthy 2017). We filtered the genotypes with VCFtools 0.1.16 (Danecek et al. 2011) to include only bi-allelic polymorphic sites with a minimal depth of six and a minimal genotype quality of 28, employing a minor allele frequency filter of 0.03 and allowing up to 20% of missing data per site. Due to high rates of missing data, two *manto* specimens were filtered out. The overall filtering resulted in 3′994 SNP sites available for our downstream analyses.

### Population genetic analyses

To infer the phylogenomic structure across all retained specimens, we first used RAxML 8.2.11 (Stamatakis 2014) implementing a generalised time-reversible (GTR) model with optimised substitution rates and a gamma model of rate heterogeneity. We further applied an ascertainment bias correction to account for the fact that we only used polymorphic SNP positions with the ASC_GTRGAMMA function implemented in RAxML. Significance was assessed using 1′000 bootstrap replicates followed by a thorough maximum likelihood search.

We inferred population structure in a first step with Admixture 1.3.0, which implements a likelihood approach to estimate ancestry (Alexander et al. 2009). We ran Admixture first across all individuals, excluding *E. eriphyle*, and then separately for *manto* and *bubastis*, to test for further intraspecific population structure. In each case, we varied the values for K, *i.e*., the number of assumed populations, from 1 to 10 and performed a cross-validation test to determine the optimal value of K. In a second step we used a principal component (PC) analysis as implemented in GenoDive 3.0.5 (Meirmans 2020) to visualize the genetic relationship among individuals. As for Admixture we performed the PC analysis including either all individuals or separately for *manto* and *bubastis*. Subsequent statistical analyses on the resulting PC scores were done using linear models in R 4.1.1 (R Core Team 2021).

Next, we estimated the level of pairwise genetic differentiation (*F*_ST_) among species and lineages using GenoDive, pooling individuals from across the distribution range for *manto* and *bubastis*, respectively. Significance was estimated based on 1′000 bootstrap iterations. We also estimated *F*_ST_ between individuals of two identified clusters within the *manto* lineage, excluding individuals that showed admixture.

Lastly, we assessed recent migration rates between species with BA3-SNPs V 3.0.4 (Mussmann et al. 2019), a modification of BayesAss (Wilson and Rannala 2003) that allows handling of large SNP datasets. First, we assessed the optimal mixing parameters for migration rates (deltaM = 0.1563), allele frequencies (delta = 0.5500), and inbreeding coefficients (deltaF = 0.0750) by running ten repetitions in BA3-SNP-autotune V 3.0.4 as recommended by Mussmann et al. (2019). Subsequently, BA3-SNPs was run with the predefined mixing parameters for 50 million generations, sampling every 100th generation. The first million generations were discarded as burn-in and chain convergence was assessed in Tracer V 1.7.1 (Rambaut et al. 2018).

## Results

Consistent with three independent taxonomic entities, the RAXML analysis resolved the *manto*, *bubastis* and *vogesiaca* lineages as distinct phylogenetic clades with 100% bootstrap support each (Fig. 2). Here, *vogesiaca* split first from the two other taxa, which formed a monophyletic group with 100% bootstrap support. Some phylogenetic structuring was observed among *bubastis* individuals, each population representing a distinct clade. In *manto,* the phylogenetic structuring was less pronounced, except for some populations; in particular, the four individuals from the easternmost part of the Swiss Alps (population 24 in Figs. 1 & 2), which constituted a sister clade to all other individuals.

**Fig. 3:**
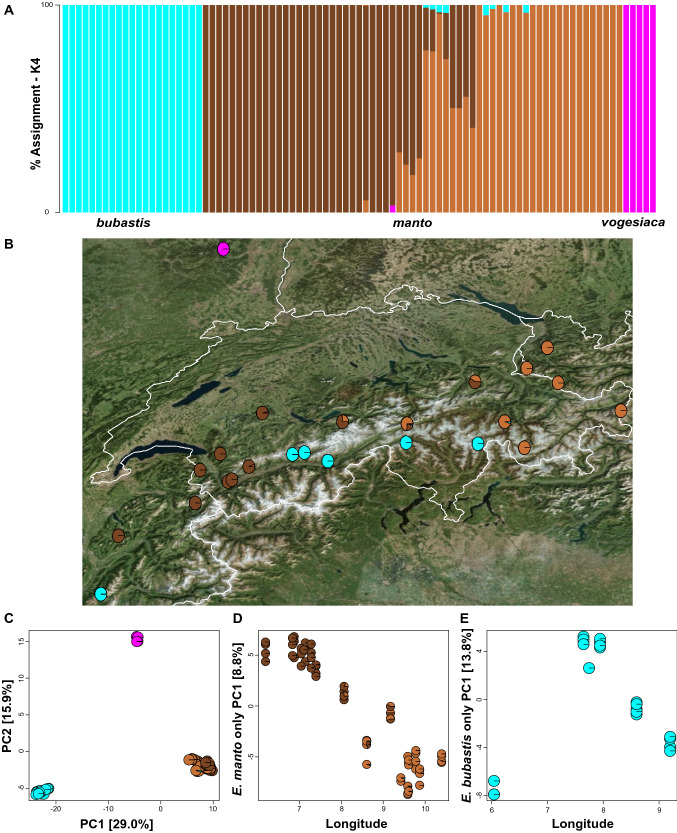
Summary of the individual based clustering analysis and their spatial relationship. **A** Individual-based assignments using Admixture for samples belonging to the *E. manto* complex. The case for four genetic clusters (K = 4) is shown as a bar diagram. **B** Map of all genotyped *E. manto* complex individuals (map modified from Bing Maps https://www.bing.com). **C** Principal component (PC) scores for all genotyped *E. manto* complex individuals. **D**–**E** Relationship between longitude and the PC1 scores for PC analyses done seperatenly on *manto* and *bubastis* individuals only. For B-E pie charts depict the Admixture assignment for each individual as shown in A

Four genetic clusters (K = 4) were the best fitting number as inferred with Admixture when *manto*, *bubastis* and *vogesiaca* were jointly analysed (Fig. 3a, b & Supplementary Information 2). Two clusters represent the *vogesiaca* and *bubastis* lineages respectively and two additional clusters occur within the *manto* lineage. The overall PC analysis suggests three main clusters corresponding to *bubastis*, *vogesiaca* and *manto* respectively, where the two *manto* clusters were grouped together (Fig. 3c). Both *bubastis* and *vogesiaca* formed a distinct genetic cluster that showed no evidence for gene flow from *manto.* Interspecific gene flow seems indeed to have primarily occurred from *bubastis* into *manto*, but its current extent seems limited (Fig. 3a). Contrasting with this result, the two genetic clusters within the *manto* lineage showed substantial genetic admixture in the central Swiss Alps (Fig. 3a, b), which was also true when Admixture was run on *manto* samples only (Supplementary Information 2). The leading axis of the PC decomposition, accounting for 8.8% of the total variation, similarly separated the two *manto* clusters, where subsequent statistical analyses showed a significant correlation between PC1 scores and longitude (*F*_1,61_ = 428.0, *p* < 0.001; *R*^2^ = 0.875; Fig. 3d). The PC1 axis for *bubastis* individuals only (Fig. 3e), accounting for 13.8% of the total variation, similarly showed a geographic clustering. However, a linear relationship between PC scores and longitude was only significant when the individuals from the French Alps were excluded (all *bubastis*: *F*_1,19_ = 0.6, *p* = 0.448; *R*^2^ = 0.031; Fig. 3e; Swiss Alps: *F*_1,16_ = 252.5, *p* < 0.001; *R*^2^ = 0.940). Despite the geographic clustering of the PC scores, Admixture did not detect any additional clusters when *bubastis* was run separately (Supplementary Information 2).

**Fig. 4:**
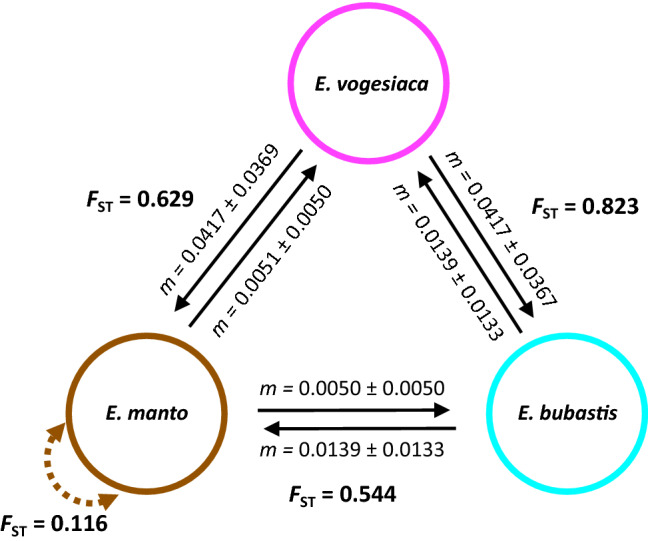
Summary of the degree of genetic differentiation (*F*_ST_) and recent migration (m) as estimated by BA3-SNPs. Black arrows indicate the direction of migration with the respective estimate of *m* ± 1 SD. For *E. manto* the *F*_ST_ between un-admixed individuals of the two inferred genomic clusters is given. All *F*_ST_ values are significant (*p* < 0.001)

The clear separation of the three lineages with very limited gene flow in Admixture is further substantiated in our assessment of contemporary migration. The latter revealed very low migration rates (*m*) between the three lineages, often being close to zero (Fig. 4). Lastly, the overall degree of genetic differentiation was substantial among our three focal lineages (*F*_ST_ *manto-bubastis* = 0.544, *F*_ST_ *manto-vogesiaca* = 0.629, *F*_ST_ *bubastis-vogesiaca* = 0.823, all *p* < 0.001; Fig. 4). This contrasts with the level of intraspecific differentiation, *i.e.* between individuals of the two *manto* clusters that showed no admixture (*F*_ST_ = 0.116, *p* < 0.001).

**Fig. 5:**
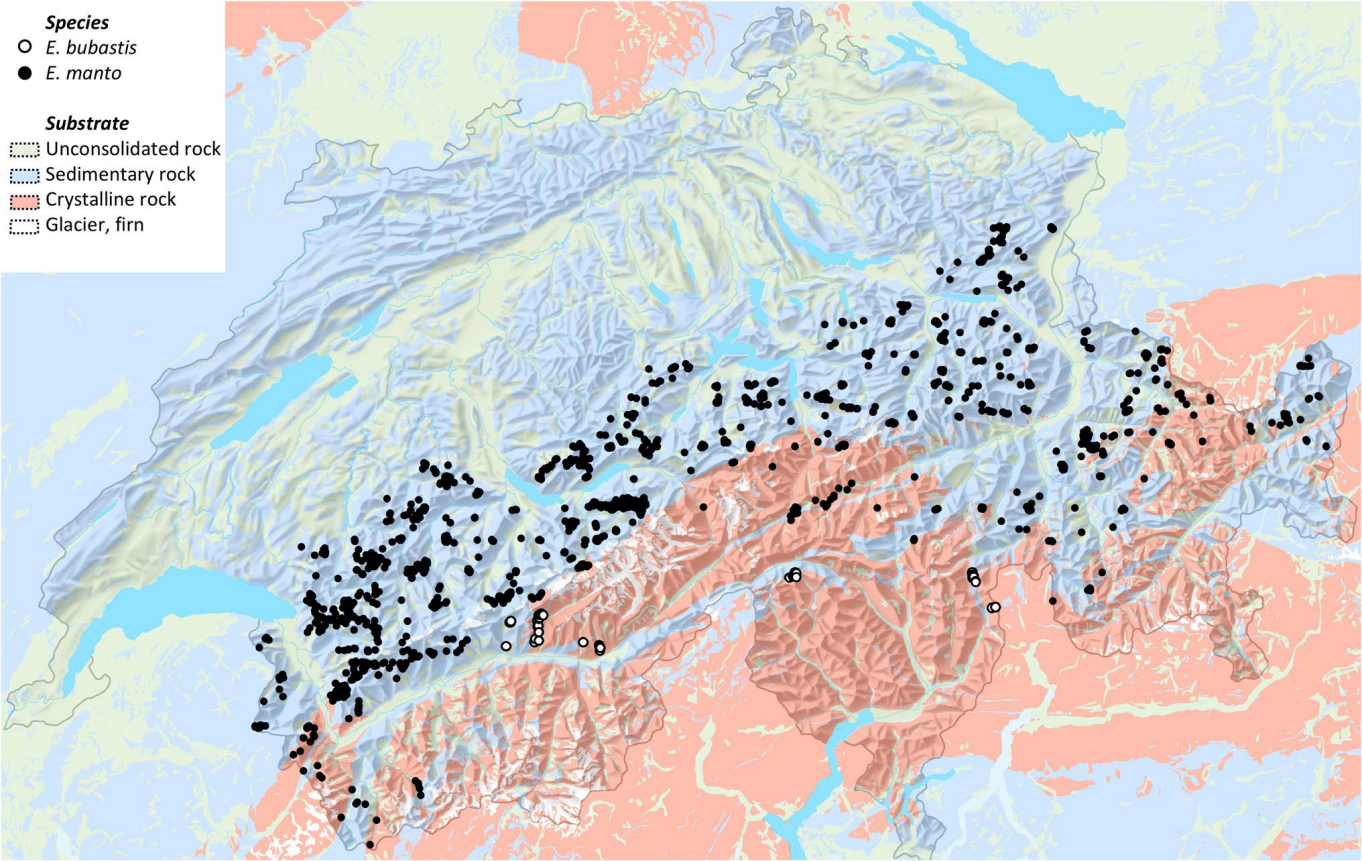
Distribution of *Erebia manto* (filled circles) and *E. bubastis* (open circles) across Switzerland. Colours indicate different major substrate types (map source: Lithological-petrographic map of Switzerland; Federal Office of Topography swisstopo, Georesources Switzerland Group)

The substrates on which *manto* and *bubastis* occurred in Switzerland differed: *manto* occurs predominantly on calcareous sedimentary rock (79.6%, N = 3'094, Fig. 5) and to a lesser degree on unconsolidated rock (14.0%, N = 543) and only rarely on siliceous crystalline rock (6.4%, N = 249). By contrast, *bubastis* did not show a preference for either siliceous crystalline (40.7%, N = 33; Fig. 5) or calcareous sedimentary rock (49.4%, N = 81); a small proportion of the observations were made on unconsolidated rock (9.9%, N = 8).

## Discussion

A species complex represents a conundrum for both taxonomy and conservation as it combines putatively cryptic species or lineages into a single taxon (Bickford et al. 2007). Classic genetic markers often do not provide the resolution to resolve such complexes (Wagner et al. 2013). Using several thousand SNPs, we examined the genetic relationships of three lineages of the *Erebia manto* complex from central Europe, *i.e.*, the *manto, bubastis* and *vogesiaca* lineages, respectively. Overall, we found that the three lineages are genetically strongly differentiated (Figs. 2, 3, 4) with very limited evidence for current gene flow (Fig. 4). Gene flow between lineages seems to have moreover occurred unidirectionally from *bubastis* into *manto* (Fig. 3). The level of genetic differentiation (*F*_ST_) between these lineages is similar to interspecific comparisons in other and taxonomically resolved *Erebia* species in the Alps (Lucek et al. 2020) and greatly exceeds differentiation within a lineage, as found for *manto* (Fig. 4). A previous study based on analyses of the same genomic dataset found that the three *E. manto* lineages have a high prevalence of the endosymbiotic bacterium *Wolbachia* and share a similar *Wolbachia* strain (Lucek et al. 2021). Given the genomic differentiation of the hosts, this could implicate that they acquired a widely distributed *Wolbachia* strain only recently. Different to other *Erebia* species (Lucek et al. 2020, 2021), *Wolbachia* seems therefore unlikely to have significantly contributed to the differentiation of the three lineages of the *manto* complex.

The strong genomic differentiation that we found between *manto* and *vogesiaca* is consistent with former genetic inferences (Schmitt et al. 2014). With a much broader sampling, the aforementioned study further recovered three distinct genetic clusters within the *manto* lineage across the Alps, which have been interpreted to originate from distinct glacial refugia. The two genetic clusters that we identified for *manto* (Fig. 3) likely correspond to the Western and the Northern clusters described by Schmitt et al. (2014). However, our denser sampling for *manto* across the Swiss Alps revealed substantial admixture between these two clusters over a relatively large geographic range (Fig. 3b). This pattern suggests that the two *manto* clusters are not reproductively isolated and thus evolutionary less differentiated than either *vogesiaca* or *bubastis* from *manto,* confirming previous hypotheses that several levels of differentiation underlie the observed variation in this complex (Cupedo 1997)*.* Inter-lineage gene flow seems to have primarily occurred from *bubastis* into *manto* (Figs. 3 & 4), which is consistent with previously reported introgression of mitochondrial haplotypes in the same direction (Cupedo and Doorenweerd 2020). Such asymmetries may occur when the degree of selection against gene flow differs between species, promoting unidirectional introgression (Pickup et al. 2019).

In Europe, the diversification of *Erebia* has been shaped by differentiation in distinct glacial refugia during the Quaternary glacial cycles, and the current distributions emerged through postglacial range expansions (Sonderegger 2005; Schmitt et al. 2006, 2016; Cupedo and Doorenweerd 2020). Distantly related *Erebia* species can often coexist and exploit different microhabitats (Kleckova et al. 2014). However, more closely related species or lineages may rather exclude each other to different degrees, which for *Erebia* falls into three broad scenarios. The first occurs when speciation is nearly completed, but rare events of introgressions may still be possible. An example is *E. nivalis*, which is found in near sympatry with *E. tyndarus* and *E. cassioides* but only exhibits very limited gene flow, possibly due to distinct chromosomal numbers, differing phenologies and micro-habitat preferences (Gratton et al. 2016; Ehl et al. 2018; Lucek et al. 2020). Other species pairs that fall under this scenario include *E. ligea* and *E. euryale* or *E. eriphyle* and *E. manto* (Sonderegger 2005; Cupedo 2014; Litman et al. 2018). The second scenario occurs in species pairs presenting a parapatric distribution, often only connected by very narrow contact zones and no morphological intergradation. This pattern is observed in *E. tyndarus* and *E. cassioides* in the Alps, both of which only meet in very restricted zones of contact (Sonderegger 2005; Lucek et al. 2020), where they form bimodal hybrid zones (Jiggins and Mallet 2000). Only rare first generation hybrids occur in such contact zones, suggesting a reduced hybrid fertility, possibly mediated by *Wolbachia*-induced incompatilibities also resulting in nearly completely interrupted gene flow (Lucek et al. 2020, 2021; Augustijnen et al. 2022). A similar situation is probably observed between *E. melampus* and *E. sudetica* in Switzerland, although no sympatric occurrence of these two taxa has been reported, at least in the Central Alps (Cupedo 1996); a genomic investigation of this complex is so far lacking (but see Haubrich and Schmitt 2007). A few additional species pairs of *Erebia* likely fall in these categories, such *E. mnestra* and *E. aethiopella* (Descimon and Mallet 2009) or *E. montana* and *E. styx* (Sonderegger 2005). The third scenario consists of more or less narrow contact zones, but not completely interrupted gene flow, leading to some morphological intergradation over a unimodal zone of secondary contact. Examples include contact zones between *E. euryale* subspecies that often have parapatric distributions, but show morphological intergradation (Sonderegger 2005; Cupedo 2014; Cupedo and Doorenweerd 2022) or between subspecies of *E. melampus* (Cupedo 1996).

Taxonomists have the somehow ungrateful task of translating this continuum into nomenclatural actions, an arbitrary, but necessary duty: delineated taxonomic units without names are *de facto* inexistant in biodiversity surveys or in conservation (Mace 2004). Current practices in Switzerland for the three scenarios outlined above mostly recognize species-level differentiations for cases that fall within the first or second scenario (SwissLepTeam 2010; Wermeille et al. 2014). The concept of "subspecies" similarly remains controversial, however, it seems to be best applied to well-delineated geographic units, either because of vicariance, or, as under the third abovementioned scenarios, narrow contact zone with limited, but existing morphological intergradation. Given our findings, the two *E. manto* lineages from the Alps, *i.e., manto* and *bubastis*, clearly fall under the second scenario given the absence of large-scale spatial overlap (Fig. 1; Sonderegger 2005) and the limited evidence for past interspecific gene flow (Figs. 3, 4).

The absence of coexistence in sympatry in the *manto* complex may on the one hand suggest that the two lineages lack enough differentiation in their ecology (Leibold and McPeek 2006) and/or phenotypic traits linked to mate choice (M’Gonigle et al. 2012) and thus occurrence in sympatry could result in interspecific mating with low offspring fitness. On the other hand, the three lineages could be ecologically differentiated and therefore exclude each other due to pronounced differences in habitat preferences. Ecological differentiation is likely limited given that in southwestern Switzerland *E. manto* and *E. bubastis* are separated by only 7–8 km (Fig. 1), without sharp geographic or climatic boundaries. Nevertheless, the vast majority of Swiss populations of *manto* seem to occur on calcareous substrate, while some populations of *bubastis* are located on or nearby siliceous substrate (Sonderegger 2005; Fig. 5). Our limited observations in France suggest that *E. manto* is similarly restricted to calcareous substrate while *E. bubastis* (at least the subspecies *willieni*) is found on siliceous substrate. Differences in geological substrate is a commonly used proxy to describe species distributions of Alpine butterflies (Illán et al. 2010; Augustijnen et al. 2022), however, what aspects of the environment may be causal in shaping the actual distributions is unknown. For example, larval development on *Poaceae* (the main host plants of *Erebia*) on specific substrates may require particular adaptations. The resulting distribution of *E. manto* and *E. bubastis* in the Alps could thus reflect the outcome of competitive exclusion to different substrates. Potential substrate association in *E. vogesiaca* requires though further investigation: while the Vosges populations are found on siliceous substrates, the possibly Jura populations are likely on calcareous substrate. In the Vosges, *E. vogesiaca* is ecologically highly specialized and restricted, living exclusively in the narrow area of the upper timberline, which in the Vosges is most formed by *Sorbus* shrubs. *E. vogesiaca* is however absent from the open pastures on the mountain ridges, as well as from the beech forests found at lower elevations.

## Conclusions and implications for conservation

Taken together, our analyses show that the three lineages of the *E. manto* complex that we studied are genetically strongly isolated, supporting their status as distinct species, in agreement with the treatment in other similar cases (Gratton et al. 2016; Lucek et al. 2020). Speciation is an evolutionary process whereby barriers to gene flow accumulate through time until gene flow becomes impossible or strongly selected against (Seehausen et al. 2014; Stankowski and Ravinet 2021). The limited and unidirectional gene flow that we observed (Fig. 3) suggests that differentiation of the three lineages characterises an advanced stage of speciation (Kulmuni et al. 2020). Such limited gene flow is also possible in other, taxonomically well resolved Alpine butterfly species (Presgraves 2002; Descimon and Mallet 2009), that in some cases have diverged since the mid-Pleistocene (Ebdon et al. 2021). Although we did not perform cross experiments, our genomic inferences provide a surrogate to estimate the potential for hybridization in the wild, showing that the latter is absent and if still possibly, likely unidirectional and selected against. Based on our findings, we therefore recommend to treat the three lineages as distinct species, especially for conservation purposes. Climate change is predicted to significantly reduce the available habitat for *E. manto* in Central Europe (Schmitt et al. 2014). Given the restricted and disjunct distributions of *E*. *bubastis* and *E*. *vogesiaca,* these species may be especially vulnerable. Indeed, some populations of both *E*. *bubastis* (Wermeille et al. 2014) and especially *E*. *vogesiaca* (IMAGO 2014) are considered to be endangered, but taxonomic uncertainties have effectively precluded conservation measures so far. Our results also suggest that future conservation measures require to integrate fine scale ecology, given the possible difference in substrates on which *E. manto* and *E. bubastis* occur. Finally, our study highlights how genomic data may be used to overcome current taxonomic uncertainties that remain in several Alpine butterflies (Litman et al. 2018). This is especially true for the genus *Erebia*, which is known for its often cryptic diversity (Sonderegger 2005), where several candidates for potentially cryptic species have been identified (Tschudin et al. 2017).

## Supplementary Information

Below is the link to the electronic supplementary material.

Supplementary file1 (XLSX 19 KB)

Supplementary file2 (DOCX 70 KB)

## Data Availability

Sequence data is available from NCBI (BioProject PRJNA909280).
