# Supplementary material for: Genomic evidence for three distinct species in the Erebia manto complex in Central Europe (Lepidoptera, Nymphalidae)
Source: Conserv Genet. 2023 Jan 10;24(3):293–304. doi: 10.1007/s10592-023-01501-w (PMC10175325; doi:10.1007/s10592-023-01501-w)
Supplement: Supplementary file 2 — Supplementary file2 (DOCX 70 KB) [file 10592_2023_1501_MOESM2_ESM.docx]

**Supplementary information of**

**Genomic evidence for three distinct species in the *Erebia manto* complex in Central Europe (Lepidoptera, Nymphalidae)**

Amanda Jospin^1^, Yannick Chittaro^2^, Daniel Bolt^3^, David Demergès^4^, Kevin Gurcel^5^, Jürgen Hensle^6^, Andreas Sanchez^2^, Christophe Praz^1,2^*, Kay Lucek^7^*^#^

^1^ Laboratory of Functional Ecology, Institute of Biology, University of Neuchâtel, Rue Emile-Argand 11, 2000, Neuchâtel, Switzerland
^2^ info fauna – CSCF, Avenue de Bellevaux 51, 2000, Neuchâtel, Switzerland

^3^ Via Crusch 8a, 7013, Domat/Ems, Switzerland

^4^ Conservatoire d'espaces naturels de Lorraine, 20 chemin de l'école des Xettes, 88400 Gérardmer, France

^5^ Allées de Sacconges 20, 74600 Seynod, France

^6^ Dorfstrasse 23, 79331 Teningen, Germany

^7^ Department of Environmental Sciences, University of Basel, Schönbeinstrasse 6, CH-4056 Basel, Switzerland

Fig. S1: Summary of the Admixture analysis using either all individuals that belong to the *manto* complex (A) or only *manto* (B) or *bubastis* individuals respectively. For each scenario the change in cross-validation (CV) error with increasing number of assumed genetic cluster (K) is shown. The best supported K is highlighted in red and the respective assignment given on the right side as barplots.
